# Inhibition of importin-7 attenuates ventilator-induced lung injury by targeting nuclear translocation of p38

**DOI:** 10.1007/s00011-023-01727-x

**Published:** 2023-04-01

**Authors:** Ning Ding, Huiqing Li, Zengzhen Zhang, Haiyan Jia

**Affiliations:** 1 Department of Anesthesiology, Shandong Provincial Third Hospital, Cheeloo College of Medicine, Shandong University, Jinan, 250031 China; 2 Department of Intensive Care Medicine, Shandong Provincial Third Hospital, Cheeloo College of Medicine, Shandong University, Jinan, 250031 China; 3 Key Laboratory of Critical Rehabilitation Medicine of Shandong Province, Shandong Provincial Third Hospital, Jinan, 250031 China

**Keywords:** Ventilator-induced lung injury, Cyclic stretch, p38 MAPK, Nuclear translocation, Importin-7

## Abstract

**Background:**

The ability of p38 to phosphorylate substrates in the nucleus and the role of nuclear p38 in the regulation of inflammation have focused attention on the subcellular localization of the kinase. Although it is clear that p38 shuttles to the nucleus upon stimulation, the mechanisms that regulate p38 nuclear input in response to mechanical stretch remain to be determined.

**Methods:**

Cyclic stretch (CS)-induced nuclear translocation of p38 was determined by Western blotting and immunofluorescence. The p38 interacting protein was identified using endogenous pull-down and protein binding assays. The potential role of importin-7 (Imp7) in CS-induced nuclear translocation of p38 and p38-dependent gene expression was confirmed using a series of in vitro and in vivo experiments. Furthermore, we tested the therapeutic potential of intratracheal administration of Imp7 siRNA-loaded nanoparticles in the ventilator-induced lung injury (VILI) mouse model.

**Results:**

We show that CS induced phosphorylation-dependent nuclear translocation of p38, which required the involvement of microtubules and dynein. Endogenous pull-down assay revealed Imp7 to be a potential p38-interacting protein, and the direct interaction between p38 and Imp7 was confirmed by in vitro and in vivo binding assays. Furthermore, silencing Imp7 inhibited CS-induced nuclear translocation of p38 and subsequent cytokine production. Notably, intratracheal administration of Imp7 siRNA nanoparticles attenuated lung inflammation and histological damage in the VILI mouse model.

**Conclusions:**

Our findings uncover a key role for Imp7 in the process of p38 nuclear import after CS stimulation and highlight the potential of preventing p38 nuclear translocation in treatment of VILI.

## Introduction

For the clinical care of critically ill patients, mechanical ventilation (MV) is a crucial tool. Despite its life-saving properties, MV can induce lung injury in the healthy lung or exacerbate acute lung injury, often known as ventilator-induced lung injury (VILI) [1]. The injurious effect of cyclic stretch (CS) on the pulmonary endothelium has been implicated in the development of VILI, which is characterized by pulmonary inflammation and particularly increased release of inflammatory mediators [2]. It has been demonstrated that modulation of ventilator settings, such as applying low tidal volumes, can reduce mortality in patients with acute respiratory distress syndrome [3]. However, the protective strategy may not meet the need in all patients, as it does not prevent pulmonary inflammation as well [4]. Therefore, it is necessary to explore the biotrauma mechanisms and additional drug therapy to target the ongoing inflammatory response in VILI.

The p38 mitogen-activated protein kinase (MAPK) pathway plays a key role in cellular responses to mechanical stresses, being involved in the regulation of inflammatory responses [5, 6]. Mechanical stress has been shown to differentially regulate gene expression, protein synthesis, and posttranslational modification and secretion of proteins and other cellular products, particularly by activating p38 signaling in alveolar epithelial cells (AECs) [7, 8]. These effects are supported by our findings that the introduction of a loss-of-function mutant of the p38 upstream kinase MAP kinase kinase 6 (MKK6) effectively inhibited CS-induced p38 activation and reduced cytokine production, confirming the importance of the p38 cascade [9]. It is well documented that p38 mediates stress signaling via dual phosphorylation of Thr180 and Tyr182 by its upstream MKK3/6 [10, 11]. Once activated, p38 exerts its functions through various downstream substrates. Phosphorylation of these substrates initiates or regulates a large number of cellular processes including transcription, translation, RNA processing, and cell cycle progression [12]. Being such a central signaling cascade, its dysregulation is associated with many pathologies, particularly inflammation [8].

The ability of p38 to phosphorylate substrates in the cytoplasm as well as in the nucleus and the role of nuclear p38 in the regulation of inflammation have diverted attention to the subcellular localization of the kinase. Early studies have shown that p38, or at least some of its isoforms, is primarily localized in the nucleus of resting cells and may be exported to the cytoplasm when stimulated [13, 14]. However, it later became clear that in most cells and conditions they were located primarily in the cytoplasm of resting cells and translocated into the nucleus upon extracellular stimulation [15–17]. This nuclear translocation is critical for cellular processes such as induction of transcription. More importantly, the nuclear shuttling regulatory mechanism has recently been used to develop a novel class of anti-inflammation drugs [18]. Compared to the inhibition of p38 signaling cascade components, the inhibition of nuclear translocation affects only a small fraction of the total p38 activity without affecting negative feedback loops; therefore, this inhibitor should be less toxic than ATP competitors that are in clinical trials [19, 20].

Despite being an essential process and a promising therapeutic target, the molecular mechanisms driving the mechanical stretch-induced nuclear translocation of p38 are not fully understood. Similar to most other members of the MAPK family, such as ERK and JNK, p38 does not contain a canonical nuclear localization signal (NLS) [19]. Furthermore, it does not appear to use passive diffusion for nuclear shuttling due to rapid nuclear translocation and the size of p38 [21]. Therefore, the stimulated nuclear translocation of p38 is likely to be regulated by a unique shuttle mechanism. Actually, several pivotal studies have reported that HSP70 [22, 23], SUMO-2 [24], as well as three β-like importins, importin 3, 7, and 9 [25] interact with p38 in response to certain stimuli, and escort the kinase into the nucleus. However, the specific protein/protein complex and the mechanisms by which these components are involved in p38 translocation in response to mechanical stretch are not clear.

In the present study, we demonstrate that CS induces phosphorylation-dependent nuclear translocation of p38, which is a microtubule- and dynein-dependent process. Using endogenous pull-down and protein binding assays, we identified importin-7 (Imp7) as a key p38 interacting protein. Importantly, Imp7 silencing inhibited CS-induced p38 nuclear import as well as cytokine release. As nuclear accumulation of p38, like other MAPKs, is responsible for the development of certain diseases [18, 26]. We therefore further tested the therapeutic potential of silencing Imp7 in the treatment of VILI. We demonstrated that intratracheal administration of Imp7 siRNA-loaded nanoparticles significantly attenuated lung inflammation and histological damage in the VILI mouse model. These findings provide new insights into the mechanism of p38 nuclear translocation and offer a basis for prospective clinical applications in VILI therapy by Imp7 inhibition.

## Materials and methods

### Animal care

Animal handling and care complied with the National Guide for Care and Use of Laboratory Animals and was approved by the ethics committee of Shandong Provincial Third Hospital affiliated to Shandong University. Animals were housed in laminar flow cages in a specific pathogen-free facility and maintained under a 12-h light–dark cycle with free access to standard laboratory chow and water. All operations were performed under anesthesia and every effort was made to minimize suffering.

### VILI model

VILI was generated in C57BL/6 mice (8–12 weeks old, 20–30 g body weight) using tidal volume MV as described previously [27]. Briefly, mice received an intraperitoneal injection of an anesthesia mixture containing ketamine (80 mg/kg) and xylazine (10 mg/kg) and were positioned supine on a heating pad. A tracheostomy was performed, after which a sterile 20-gauge catheter was inserted into the trachea and sutured to avoid air leakage. Mice were then connected to a small animal ventilator (Inspira; Harvard Apparatus, Holliston, MA, USA) and ventilated for 4 h in volume control mode with a tidal volume of 15 ml/kg and a respiratory rate of 100 breaths/min. The positive end air pressure was zero and the inspired oxygen fraction was 0.21. The ventilation parameters were chosen based on results from preliminary experiments showing that these conditions lead to VILI in mice with reproducible alterations in lung inflammation. Control animals were anesthetized and underwent tracheostomy but breathed spontaneously. During the experiment, mice were injected intraperitoneally with ketamine and xylazine at 45-min intervals at one-third of the initial dose to maintain anesthesia, and intraperitoneal injection of saline (0.01 ml/g body weight) to maintain intravascular volume status. After the experiment was completed, the mice were euthanized with 150 mg/kg ketamine intraperitoneally. Lung tissue was removed for wet/dry weight ratio assessment, Western blotting, and myeloperoxidase (MPO) activity assay, and bronchoalveolar lavage fluid (BALF) was collected for cytokine analysis. Separate animals were used for histology and immunofluorescence (n = 3).

### Evaluation of lung inflammation and histological injury

The levels of tumor necrosis factor-α (TNF-α), interleukin-1β (IL-1β), and IL-6 in BALF were determined by ELISA according to the manufacturer’s instructions (R&D Systems, Minneapolis, MN, USA). As a marker for neutrophil infiltration, MPO activity in lung tissue lysates was determined with a mouse MPO ELISA kit (jiancheng, Nanjing, China) according to the manufacturer’s protocol. Lung wet weight was determined immediately after removal, then the lungs were placed in an oven at 75 °C for 48 h and reweighed. The wet/dry weight ratio was calculated as the ratio of wet weight to dry weight. For histological examination, lung tissue was serially sectioned, embedded in paraffin, and stained with hematoxylin and eosin (H&E).

### Primary AECs isolation

Primary type II AECs were isolated from male Sprague–Dawley rats and cultured as previously described [28, 29]. Briefly, rat lungs were isolated in a sterile site. Protease solution (300 U/ml collagenase type I, 4 U/ml elastase, 5 U/ml dispase, and 100 μg/ml DNase I in Hanks’ balanced salt solution) was used for the digestion of the lungs for 25 min at 37 °C. Next, a continuous digestion with 0.1% trypsin–EDTA and 100 μg/ml DNase I was performed for 20 min at 37 °C. Digested lungs were dissociated and made into a single cell suspension. Purity was assessed using flow cytometry. AECs were seeded onto culture dishes at 1 × 10^6^/cm^2^ and cultured in 5% CO_2_ and 95% air in Dulbecco’s modified Eagle’s medium (DMEM) containing 10% fetal bovine serum, 2 mM l-glutamine, 100 U/ml penicillin, and 0.1 mg/ml streptomycin. Experiments were conducted the day after isolation.

### Cyclic stretch

AECs were seeded on collagen I-coated BioFlex culture plates (Flexercell International, McKeesport, PA, USA) at a density of 1.2 × 10^5^ cells/well. Plates were mounted on a Flexercell Tension Plus system (FX-4000T; Flexercell International, McKeesport, PA, USA). This system provides uniform radial and circumferential cyclic strain on the membrane surface along all radii, mimicking the lung breathing mechanisms [30]. According to preliminary data, cells undergo 15% elongation at 24 cycles per minute for various time points with the stretch/relax ratio of 1:1, which mimics the mechanical stress induced by high tidal volume MV [30, 31]. Control BioFlex plates with static cell culture were placed in the same cell culture incubator. When necessary, cells were preincubated for 1 h before CS with either gossypetin (60 µM; ChromaDex Inc., St. Santa Ana, CA, USA), SB203580 (5 µM; Sigma-Aldrich, St. Louis, MO, USA), nocodazole (1 µM; Calbiochem, San Diego, CA, USA), paclitaxel (100 nM; Sigma-Aldrich, St. Louis, MO, USA), cytochalasin D (1 µM; Sigma-Aldrich, St. Louis, MO, USA), phalloidin (10 µM; Sigma-Aldrich, St. Louis, MO, USA), ciliobrevin D (20 µM; EMD Millipore, Billerica, MA), ML-7 (10 µM; Sigma-Aldrich, St. Louis, MO, USA), or vehicle control DMSO (Sigma-Aldrich, St. Louis, MO, USA).

### Western blotting

Cells were washed twice in phosphate-buffered saline (PBS) and lysed with 1 × radio-immunoprecipitation assay (RIPA) lysis buffer (Beyotime, Shanghai, China) for 10 min on ice. Lung samples were homogenized in 1 × RIPA lysis buffer. The protein content of cell lysates was determined by bicinchoninic acid (BCA) assay (Beyotime, Shanghai, China). For Western blotting, equal amounts of proteins were separated by SDS-PAGE gel electrophoresis and transferred to nitrocellulose membranes followed by immunostaining with primary antibody specific for p38 (1:1000), phosphorylated p38 (p-p38) (1:1000), ATF-2 (1:1000), p-ATF-2 (1:1000), p-MK2 (1:1000), p-Elk1 (1:1000) (Cell Signaling Technology, Danvers, MA, USA), and Imp7 (1:1000, Abcam, Cambridge, MA, USA). A horseradish peroxidase-conjugated secondary antibody (1:2000, Pierce, Rockford, IL, USA) was then used in a standard enhanced chemiluminescence reaction according to the manufacturer’s instructions. In some experiments, Western blotting was used to detect the Imp7 proteins extracted from the cytoplasm and nucleus with NE-PER nuclear and cytoplasmic extraction reagents (Pierce Thermo Scientific, Rockford, IL, USA).

### Immunofluorescence

Cells were fixed with 2% (w/v) paraformaldehyde, permeabilized with 0.1% Triton X, and blocked with 2% bovine serum albumin (BSA) in PBS. Lung tissue sections were deparaffinized, permeabilized with 0.1% Triton X-100, and blocked with 5% BSA. The samples were then incubated with primary antibody specific for p38 (1:500), p-p38 (1:200), p-MK2 (1:200), and p-Elk1 (1:200) (Cell Signaling Technology, Danvers, MA, USA), Imp7 (1:200, Thermo Fisher Scientific, Rockford, IL, USA), and F-actin (1:500, Invitrogen, San Diego, CA, USA). Samples were washed with PBS containing 0.5% BSA followed by incubation in appropriate Cy3 (1:1000, Invitrogen, Carlsbad, CA, USA) and Cy5 (1:1000, Jackson ImmunoResearch Laboratories, West Grove, PA, USA) conjugated secondary antibodies, and then stained with 100 ng/ml DAPI (Sigma-Aldrich, St. Louis, MO, USA) to visualize nuclei. Positively stained cells in 6 random fields were imaged with a confocal microscope (FluoView 1000; Olympus, Melville, NY, USA). For quantification, RGB images were adjusted to the maximum entropy threshold and analyzed using Image J software (NIH Image, Bethesda, MD, USA).

### Real-time quantitative PCR

Total RNA was extracted from cell samples using the RNeasy Mini Kit (Qiagen, Valencia, CA, USA). For each sample, 1 μg RNA was reverse transcribed using the iScript reverse transcription supermix kit (Bio-Rad, Hercules, CA, USA). PCR amplification mixtures were prepared using iTaqTM Fast SYBR Green Supermix with ROX (Bio-Rad, Hercules, CA, USA) and Real-time PCR was performed with MX3000p (Stratagene, La Jolla, CA, USA). Quantification of TNF-α, IL-1β, and IL-6 gene expression was normalized to endogenous β-actin.

### Pull-down assay

CS-stimulated cells and static controls were lysed as described above. The samples (200 μg protein) were then incubated with Sepharose beads-conjugated p-p38 antibody (Cell Signaling Technology, Danvers, MA, USA) in reaction buffer (50 mM Tris–HCl pH 7.5, 5 mM EDTA, 150 mM NaCl, 1 mM DTT, 0.01% NP40, 2 μg/mL BSA, 0.02 mM PMSF, 1 × protease inhibitor mixture). After overnight incubation at 4 °C with gentle shaking, the beads were washed 5 times with buffer (50 mM Tris–HCl pH 7.5, 5 mM EDTA, 150 mM NaCl, 1 mM DTT, 0.01% NP40, 0.02 mM PMSF), and proteins bound to the beads were analyzed by Western blotting.

### In vitro binding assay

GST and GST-Imp7 were purified with Glutathione Sepharose 4B beads (GE Healthcare, Pittsburg, PA, USA) and His-p38 protein was purified with Ni^2+^-NTA agarose (Qiagen, Chatsworth, CA, USA) following the manufacturers’ protocols. His-p-p38, for the in vitro Imp7 pull-down assay, was obtained by incubating His-p38 with an ATP regeneration system [Ojala PM, Sodeik B, Ebersold MW, Kutay U, Helenius A. Herpes simplex virus type 1 entry into host cells: reconstitution of capsid binding and uncoating at the nuclear pore complex in vitro. Mol Cell Biol. 2000;20(13):4922–4931.]. Equal amounts of GST and GST-Imp7 were incubated with His-p38 or His-p-p38 in protein binding buffer (50 mM Tris–HCl pH 8.0, 1 mM EDTA, 100 mM NaCl, 1% Triton X-100, 5 mM DTT) for 3 h on a rotary shaker at 4 °C. After 3 washes with binding buffer, the beads were boiled in 1 × SDS loading buffer (50 mM Tris–HCl pH 6.8, 2% SDS, 0.1% bromophenol blue, 10% glycerol, 100 mM DTT) and then proceeded to SDS-PAGE.

### Co-immunoprecipitation

Cells were washed with ice-cold PBS and then lysed with IP lysis buffer (Pierce, Rockford, IL, USA). Whole cell lysates were collected and centrifuged at 10,000×*g* for 10 min at 4 °C. The resulting supernatant was quantified using the BCA assay. For co-immunoprecipitation with transfected cells, 500 μg of total protein was used to incubate with anti-FLAG conjugated Sepharose beads (Sigma-Aldrich, St. Louis, MO, USA) for 4 h on a rotating rocker at 4 °C. For endogenous co-immunoprecipitation with anti-Imp7 antibody, 1 mg of total protein was used. Immunocomplexes bound to the Sepharose beads were recovered by brief centrifugation followed by 3 washes with cold PBS. The harvested beads were suspended in 20 μl of 5 × SDS-PAGE sample buffer and boiled for 5 min. A 50-μg cell lysate was used as an input control. Samples were analyzed by Western blotting.

### Proximity ligation assay (PLA)

In situ PLA was used to simultaneously detect p38 and Imp7 upon spatial proximity in CS-stimulated AECs. Cells were fixed with 4% paraformaldehyde and permeabilized with 0.3% Nonidet P (NP)-40. PLA was performed using the Duolink PLA kit (Sigma-Aldrich, St. Louis, MO, USA) according to the manufacturer’s instructions. Briefly, fixed cells were incubated overnight with anti-p-p38 and anti-Imp7 antibodies. After multiple washes, cells were sequentially incubated with PLA probe, ligation solution, and amplification solution at 37 °C. The pictures were taken with a confocal microscope (FluoView 1000; Olympus, Melville, NY, USA).

### siRNA transfections and rescue experiments

siRNAs targeting Imp7, Imp-α1, Imp-α5, Imp-β1, Imp3, Imp9, p150Glued (the main component of the dynein-dynactin complex), MLCK or a scramble control (control siRNA) were obtained from Dharmacon (Lafayette, CO, USA). siRNAs were transfected at 20 nM with Lipofectamine RNAiMAX (Thermo Fisher Scientific, Waltham, MA, USA). For rescue experiments, Imp7 siRNA was mixed with HA-Imp7 plasmid and transfected with Lipofectamine 2000 (Thermo Fisher Scientific, Waltham, MA, USA). siRNA/plasmid complexes were suspended in Opti-MEM (Thermo Fisher Scientific, Waltham, MA, USA) and incubated with transfection reagent for 15 min at room temperature. The mixture was then incubated with AECs in serum- and antibiotic-free conditions for 12 h at 37 °C. After the cells were washed twice with sterile PBS, they were incubated for an additional 12 h before exposure to CS.

### Preparation of Imp7 siRNA-loaded nanoparticles

Imp7 siRNA-encapsulated nanoparticles were prepared as previous reported [32]. Briefly, Imp7 siRNA was dissolved in citrate buffer (10 mM, pH 3.0) and quickly mixed with the lipid mixture by vortexing. The lipid mixture consisted of C12-200, cholesterol, DSPC, and mPEG-DMG dissolved in ethanol at a molar ratio of 50:38.5:10:1.5. Unentrapped siRNA was removed by ultrafiltration centrifugation. Entrapment efficiency was measured by RiboGreen assay (Molecular Probes, Eugene, OR, USA). The siRNA-loaded nanoparticles were diluted in PBS for in vivo therapeutic studies. For the biodistribution study, DiR-labeled Imp7 siRNA nanoparticles were prepared by dissolving DiR at 1% w/w of total lipids in an alcoholic aqueous phase. Mice were anesthetized and injected intratracheally with 500 µL of DiR-labeled nanoparticles in PBS. Whole body images of animals were captured by the IVIS Lumina XR in vivo imaging system (Caliper Life Sciences, Hopkinton, MA, USA) using excitation/emission wavelengths of 740/790 nm. Untreated animals were used as controls.

### Statistical analysis

All statistical analyses were performed using SigmaPlot 14.0 (Systat Software, Point Richmond, CA, USA). Data are expressed as mean ± SEM. Comparisons between two groups under the same treatment were performed by Student’s *t* test. We applied Welch’s test when statistical heterogeneity was evident. Significance was established at *P* < 0.05. P values were indicated by asterisks as followed: *P < 0.05, **P < 0.01, and ***P < 0.001.

## Results

### Cyclic stretch-induced nuclear translocation of p38

**Fig. 1:**
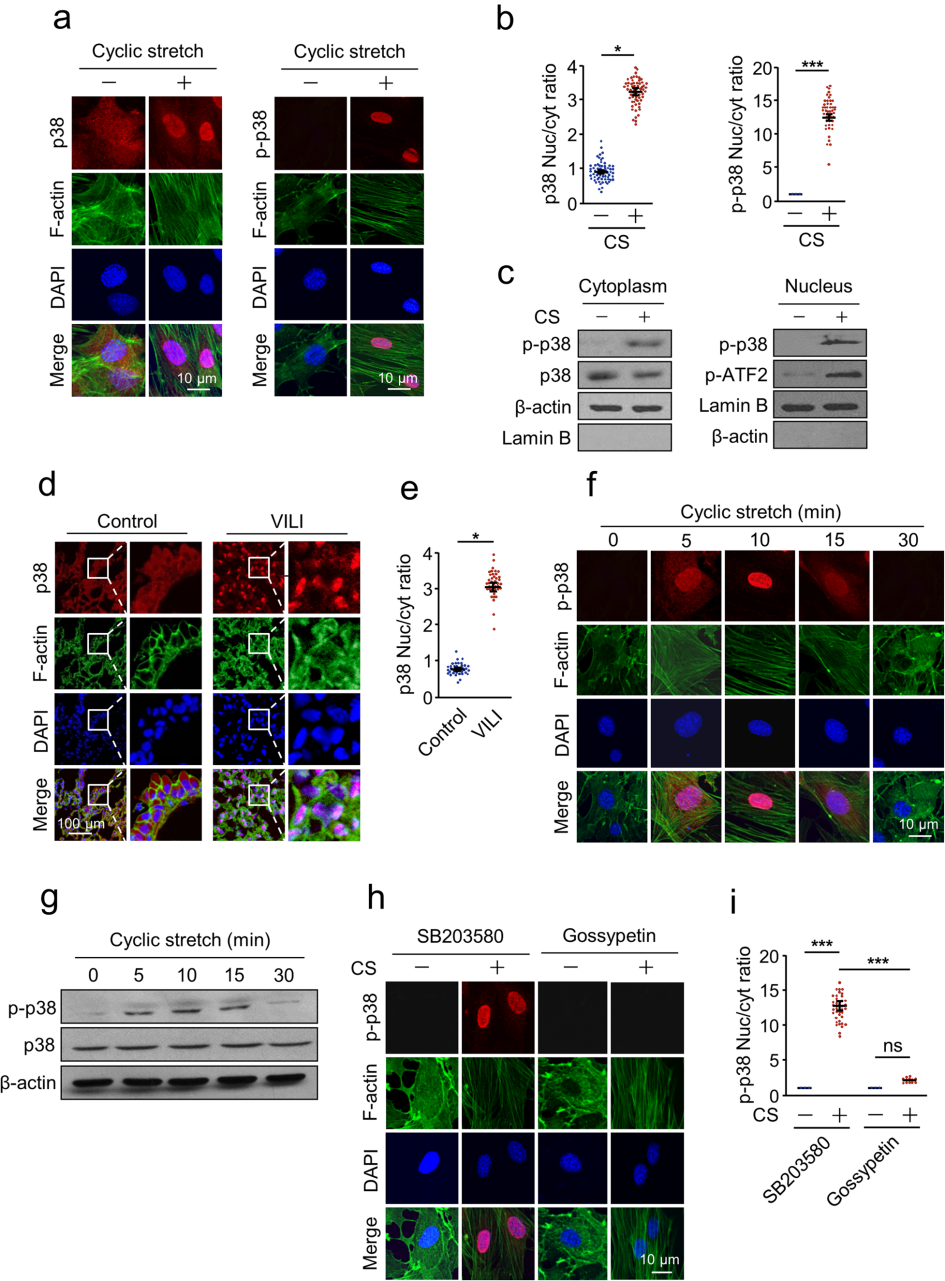
Cyclic stretch induced the nuclear translocation of p38. **a**, **b** Nuclear translocation of total or phosphorylated p38 (p-p38) induced by cyclic stretch (CS). **a** AECs were treated with or without CS for 10 min, and immunostaining was performed with p38/p-p38 (red), F-actin (green) antibodies, and counterstained with DAPI to detect nuclei (blue). **b** Quantification of the nucleo-cytoplasmic distribution of p38/p-p38. **c** Cell fractionation analyses of CS-induced nuclear translocation of p38. AECs were treated with or without CS for 10 min, followed by nuclear and cytoplasmic extraction. Western blotting was performed with p-p38, p38, p-ATF2, β-actin, and Lamin B antibodies. **d**, **e** Nuclear translocation of p38 induced by high tidal volume ventilation in a mouse model of ventilator-induced lung injury (VILI). **d** The animals were ventilated for 4 h with a tidal volume of 15 ml/kg and a respiratory rate of 100 breaths/min, and immunostaining was performed with p38 (red), F-actin (green) antibodies, and counterstained with DAPI to detect nuclei (blue). **e** Quantification of the nucleo-cytoplasmic distribution of p38. **f** The time course of nuclear translocation of p38 induced by CS. AECs were treated with CS for the indicated time. Immunostaining was performed with p38 (red), F-actin (green) antibodies, and counterstained with DAPI to detect nuclei (blue). **g** The time course of nuclear translocation of p38 induced by CS. AECs were treated with CS for the indicated time. Western blotting was performed with p-p38, p38, and β-actin antibodies. **h**, **i** The effect of p38 phosphorylation on its nuclear translocation. AECs were preincubated with p38 specific inhibitor SB203580 (5 µM), MKK3/6 inhibitor gossypetin (60 µM), and treated with or without CS for 10 min. **h** Immunostaining was performed with p-p38 (red), F-actin (green) antibodies, and counterstained with DAPI to detect nuclei (blue). **i** Quantification of the nucleo-cytoplasmic distribution of p-p38. Three independent experiments were analyzed. Significance: *P < 0.05, ***P < 0.001, *NS* no significance; two-tailed Student’s *t* tests. (color figure online)

To determine whether mechanical stretch induces the nuclear translocation of p38, we established an in vitro model of cell cyclic stretch and visualized the intracellular localization of total p38 and p-p38 by immunofluorescence. The results showed that in resting cells, p38 was dispersed in the whole cell (Fig. 1a, b). Treatment of AECs with CS resulted in significant nuclear accumulation of p38. For p-p38, the intensity and distribution of fluorescence showed not only significant activation of p38 but also nuclear localization of p-p38 on CS (Fig. 1a, b). Western blotting of cytoplasmic and nuclear extracts confirmed the results of immunostaining. Under CS challenge, both p38 and its substrate ATF-2 were activated (Fig. 1c). Consistent with the in vitro findings, nuclear accumulation of p38 was also observed in a mouse model of VILI (Fig. 1d, e). Together, these data demonstrate that mechanical stretch induces nuclear translocation of p38 in vitro and in vivo.

We next determined the time course of CS-induced p38 nuclear import. The results showed that CS induced rapid nuclear accumulation of p38 within 5 min. The accumulation peaked at 10 min, decreased gradually, and returned to resting levels by 30 min (Fig. 1f). Since the time course is similar to that of p38 activation (Fig. 1g), it seems that CS-induced nuclear translocation of p38 is a phosphorylation-related process. To further examine the effect of p38 phosphorylation on its nuclear translocation, we used the MKK3/6 inhibitor gossypetin (which inhibits p38 phosphorylation by directly inhibiting MKK3/6 kinase activity) and the widely used p38-specific inhibitor SB203580 (block the kinase activity of p38, but not its phosphorylation) to treat the cells. Immunofluorescence assay confirmed that SB203580 pretreatment had no effect on CS-induced nuclear accumulation of p38, however, p38 failed to translocate into the nucleus in gossypetin treated cells (Fig. 1h, i). These data suggest that phosphorylation of p38, but not its kinase activity, plays a critical role in determining its intracellular localization.

### The nuclear translocation of p38 is microtubule- and dynein-dependent

**Fig. 2:**
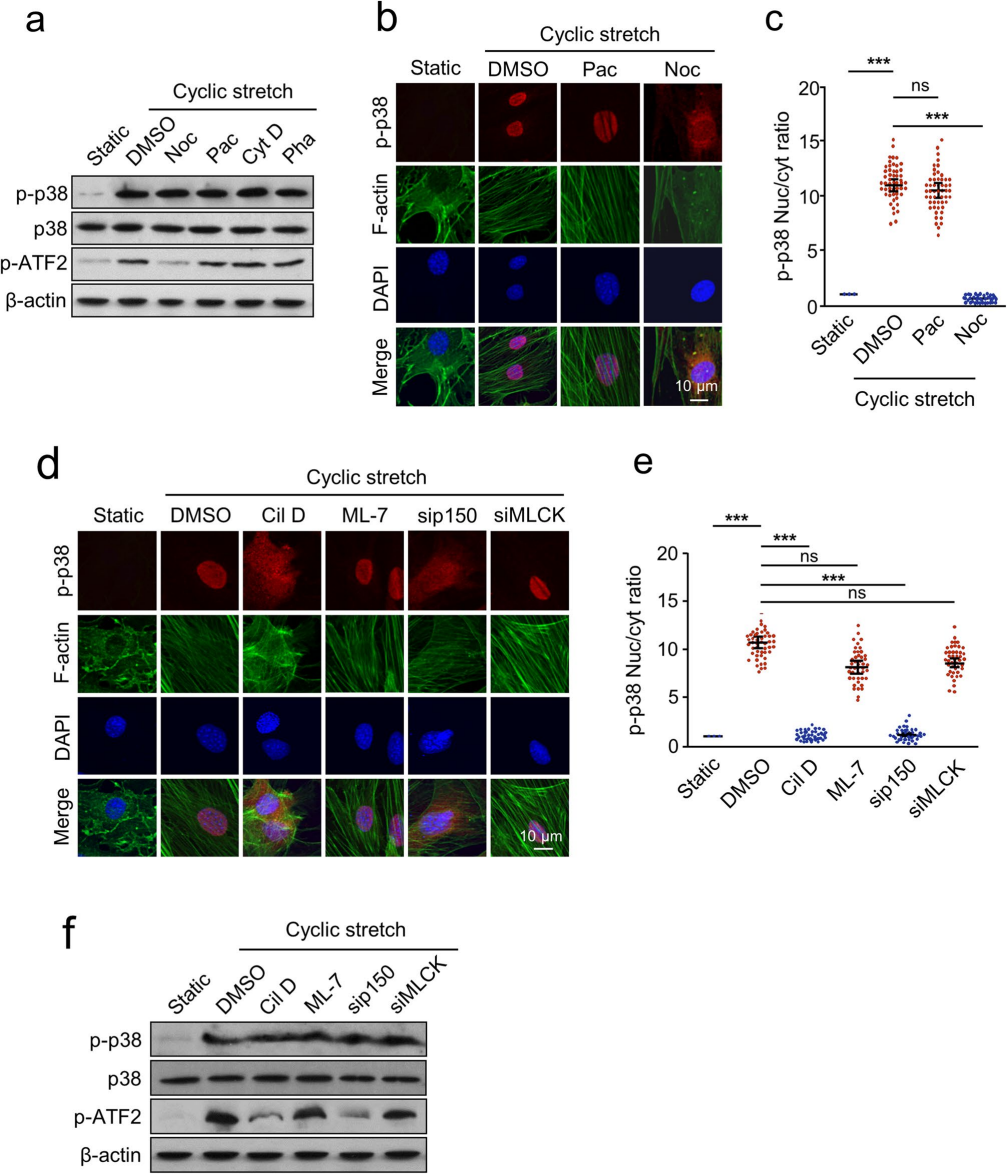
Microtubule and dynein are required for the cyclic stretch (CS) induced nuclear translocation of p38. **a** AECs were preincubated with or without nocodazole (Noc, 1 µM), paclitaxel (Pac, 100 nM), cytochalasin D (Cyt D, 1 µM), or phalloidin (Pha, 10 µM), and treated with CS for 10 min, then Western blotting was performed with p-p38, p38, p-ATF2, and β-actin antibodies. **b**, **c** AECs were preincubated with paclitaxel (Pac, 100 nM) or nocodazole (Noc, 1 µM), and treated with CS for 10 min. **b** Immunostaining was performed with p-p38 (red), F-actin (green) antibodies, and counterstained with DAPI to detect nuclei (blue). **c** Quantification of the nucleo-cytoplasmic distribution of p-p38. **d**, **e** AECs were preincubated with or without ciliobrevin D (Cil D, 20 µM), ML-7 (10 µM), p150Glued siRNA (sip150), MLCK siRNA (siMLCK), and treated with CS for 10 min. **d** Immunostaining was performed with p-p38 (red), F-actin (green) antibodies, and counterstained with DAPI to detect nuclei (blue). **e** Quantification of the nucleo-cytoplasmic distribution of p-p38. **f** AECs were preincubated with or without the chemicals described above, and treated with CS for 10 min, and Western blotting was performed with p-p38, p38, p-ATF2, and β-actin antibodies. Cells that were not subjected to CS served as a negative control (static). Three independent experiments were analyzed. Significance: ***P < 0.001, *NS* no significance; two-tailed Student’s *t* tests. (color figure online)

To address whether microtubules and/or microfilaments play a role in the efficient delivery of p38 to the nucleus, AECs were incubated for 2 h prior to and during CS with the microtubule-depolymerizing reagent nocodazole, the microtubule-stabilizing reagent paclitaxel, the microfilament assembly-destroying reagent cytochalasin D, and the microfilament-stabilizing reagent phalloidin. Phosphorylation of p38 and its substrate ATF-2 was measured by Western blotting. As shown in Fig. 2a, none of the reagents blocked p38 phosphorylation. Interestingly, only nocodazole pretreatment blocked ATF-2 phosphorylation. The phenomenon was independently confirmed by immunofluorescence with p-p38 staining. The results showed that nocodazole also blocked CS-induced nuclear translocation of p38 (Fig. 2b, c). These findings suggest that microtubules do not affect the phosphorylation of p38, but are critical for its nuclear import in response to CS and subsequent phosphorylation of ATF-2.

We next investigated the effect of the motor protein dynein on CS-induced p38 nuclear import. The nuclear import was almost completely blocked by pretreatment with the dynein inhibitor ciliobrevin D, whereas the MLCK inhibitor ML-7 did not affect this process (Fig. 2d, e). The results were further confirmed by siRNA transfection experiment that CS-induced nuclear translocation of p38 was significantly inhibited in cells transfected with p150^Glued^ siRNA, but not MLCK siRNA (Fig. 2d, e). Consistently, pretreatment with ciliobrevin D or transfection with p150^Glued^ siRNA also showed an inhibitory effect on ATF-2 phosphorylation (Fig. 2f). Collectively, these results suggest that CS-induced nuclear translocation of p38 is an energy-consuming process that relies primarily on microtubule-based motors.

### Pull-down assay reveals Imp7 is a critical p38 binding protein

**Fig. 3:**
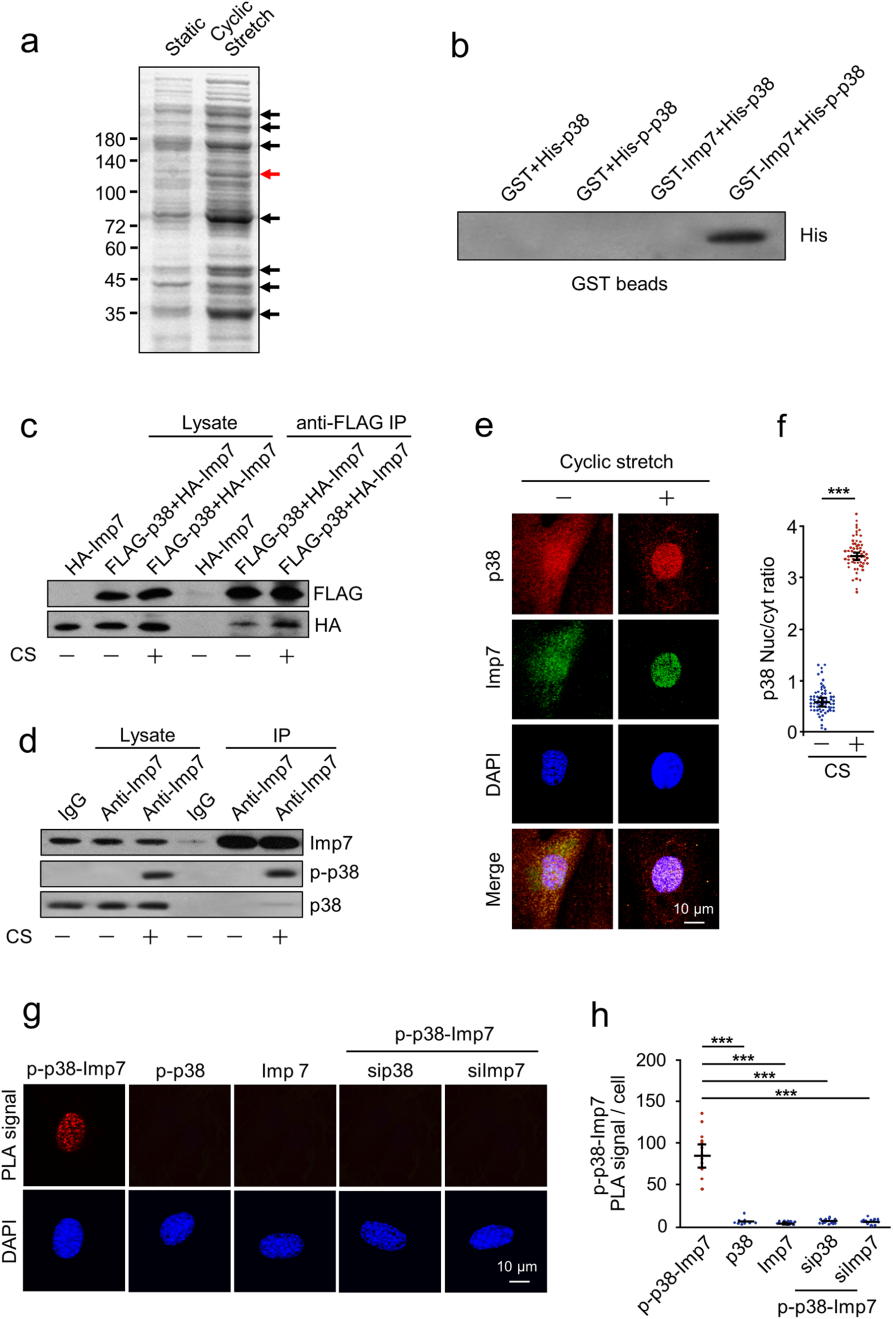
Cyclic stretch-induced interaction between p38 and Imp7. **a** AECs were treated with or without CS, lysed, and incubated with a Sepharose bead-conjugated p-p38 antibody. The proteins bound to the beads were subjected to SDS-PAGE. The arrows indicate notable bands that co-immunoprecipitated in CS-induced AECs. **b** GST-tagged Imp7 and GST were purified and used for an in vitro binding assay with His-tagged p-p38 bound Ni^2+^-NTA Resin. **c** AECs were either transfected with HA-Imp7 alone, or co-transfected with FLAG-p38 and HA-Imp7 and treated with or without CS. Western blotting of whole cell extracts and immunoprecipitates were performed using anti-FLAG or anti-HA antibodies. **d** AECs were treated with or without CS, then immunoprecipitation with Imp7 antibody was performed. Western blotting of whole cell extracts and immunoprecipitates were performed using anti-Imp7, anti-p-p38 or anti-p38 antibodies. **e**, **f** AECs were treated with or without CS and double-stained with primary antibodies against p38 (red) or Imp7 (green), followed by incubation with Cy3/Cy5-conjugated secondary antibodies, respectively. The nuclei of cells were stained with DAPI (blue). **f** Quantification of the nucleo-cytoplasmic distribution of p38. **g**, **h** In situ PLA detection of the association between endogenous p38 and Imp7 in AECs. Red dots represent PLA events and indicate the close proximity between p38 and Imp7 proteins. Cell nuclei are stained in blue with DAPI. **h** Quantification of the PLA signal. Three independent experiments were analyzed. Significance: ***P < 0.001; two-tailed Student’s *t* tests. (color figure online)

To explore the potential p38 binding proteins, we performed an endogenous pull-down assay using Sepharose bead-conjugated p-p38 antibody. We observed some prominent bands co-immunoprecipitated in CS-stimulated cells compared with static cells (indicated by arrows, Fig. 3a). The bands were cut and identified by mass spectrometry. Among the identified proteins, Imp7, a 120 kDa nuclear transport receptor (NTR), was selected for the next study (indicated by red arrow, Fig. 3a). This is because Imp7 has recently been reported to play an important role in the nuclear import of activated MAPKs in Drosophila and vertebrates [18, 33, 34]. Therefore, we hypothesized that Imp7 could regulate p38 nuclear import in response to mechanical stretch.

To confirm the results of mass spectrometry, we first performed a GST pull-down analysis to demonstrate that Imp7 and p-p38 interact directly in vitro*.* GST-Imp7, but not control GST, bound to p-p38, indicating a direct interaction between Imp7 and p-p38 (Fig. 3b). Next, co-immunoprecipitation of FLAG-p38 with HA-Imp7 was performed in live cells. HA-Imp7 was co-precipitated by FLAG-p38, especially in response to CS (Fig. 3c). We then used an antibody that recognizes Imp7 to investigate the association of endogenous p-p38 with Imp7 in cells. Immunoprecipitation of Imp7 efficiently co-immunoprecipitated endogenous p-p38 from cell lysates; however, a control antibody IgG did not (Fig. 3d). This result was further validated by analyzing the co-localization of p-p38 and Imp7. Immunofluorescence staining showed that both p38 and Imp7 were dispersed in static cells. While both proteins translocated into the nucleus in response to CS, showing a prominent co-localization (Fig. 3e, f).

As an independent test of association, we performed the PLA experiment, a method to detect in situ protein interactions with high specificity and sensitivity [35]. Staining of p-p38 and Imp7 has shown to produce strong PLA signals, whereas any antibody alone gave no signal (Fig. 3g, h). As expected, p-p38-Imp7 PLA signals were detected in the nucleus. Importantly, knockdown of p38 or Imp7 resulted in a significant decrease in PLA signal, validating the specificity of the assay (Fig. 3f, g). Thus, the association measured by PLA represents a specific p-p38-Imp7 interaction in living cells.

### Imp7 is required for CS-induced nuclear translocation of p38

**Fig. 4:**
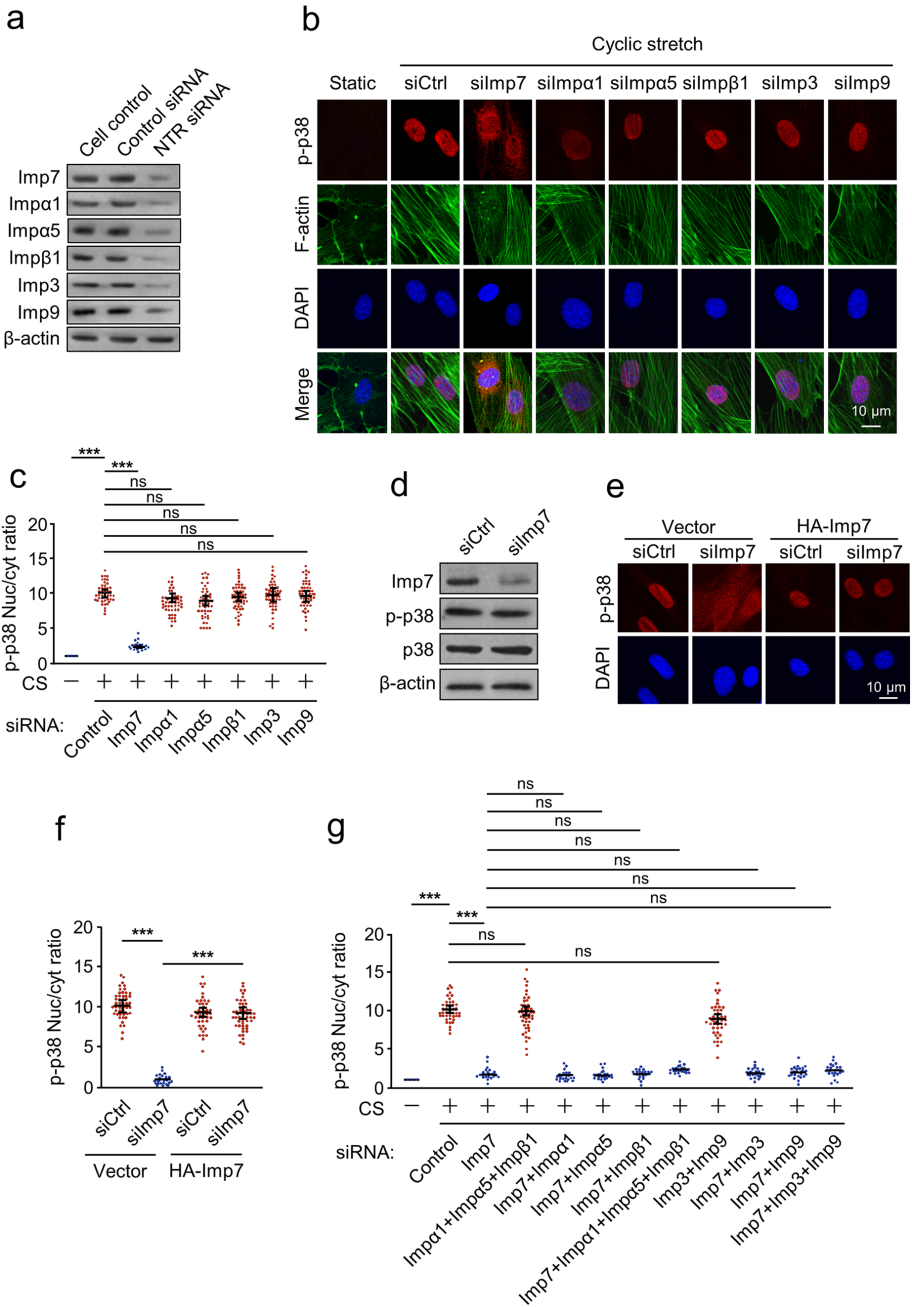
Imp7 is required for CS-induced nuclear translocation of p38. **a** Knockdown efficiency for NTR siRNA verified by Western blotting. **b**, **c** AECs were transfected with siRNA specific to Imp7 (siImp7), Imp-α1 (siImpα1), Imp-α5 (siImpα5), Imp-β1 (siImpβ1), Imp3 (siImp3), Imp9 (siImp9), or control (siCtrl), respectively, and treated with CS for 10 min. **b** Immunostaining was performed with p-p38 (red), F-actin (green) antibodies, and counterstained with DAPI to detect nuclei (blue). Cells that were not subjected to CS served as a negative control (static). **c** Quantification of the nucleo-cytoplasmic distribution of p-p38. **d** AECs were preincubated with siImp7 or siCtrl, and treated with CS for 10 min, then Western blotting was performed with Imp7, p-p38, p38, and β-actin antibodies. **e**, **f** Immunofluorescence of p-p38 localization in AECs silenced with siImp7. An empty vector or HA-Imp7 was overexpressed. Cell nuclei are stained in blue with DAPI. **f** Quantification of the nucleo-cytoplasmic distribution of p-p38. **g** Quantification of the effect on CS-induced p38 nucleo-cytoplasmic distribution upon silencing of the indicated importins. Three independent experiments were analyzed. Significance: ***P < 0.001, *NS* no significance; two-tailed Student’s *t* tests. (color figure online)

Next, we explored the role of Imp7 in CS-induced nuclear translocation of p38. We downregulated Imp7 by specific siRNA in AECs and stained for p38. siRNAs targeting other well-characterized NTRs (Imp3, Imp9, Imp-α1, Imp-α5, and Imp-β1) were also used to further examine whether the nuclear translocation of p38 and its role on nuclear targets were shared by other proteins of the nucleo-cytoplasmic transport machinery. The knockdown efficiency for siRNA was confirmed by Western blotting (Fig. 4a). Immunostaining results showed that Imp7 depletion significantly inhibited p38 nuclear import. Contrary to Imp7, none of the other NTRs knockdown inhibited p38 nuclear translocation (Fig. 4b, c). In contrast, p-p38 protein level was not affected by siImp7 (Fig. 4d). Rescue of Imp7 expression in siImp7-transfected cells almost completely restored nuclear p38, indicating the effect of siImp7 on p38 re-localization was specific (Fig. 4e, f). Thus, Imp7, but not other NTRs, is required for CS-induced p38 nuclear import.

To further determine whether there is a synergistic action of Imp7 with other NTRs, we downregulated these NTRs in different combinations. The results showed that simultaneous silencing of Imp7 and each NTR, triple knockdown of Imp3, Imp9 and Imp7, and quadruple knockdown of Imp-α1, Imp-α5, Imp-β1 and Imp7 did not improve the effect obtained by silencing Imp7 alone (Fig. 4g), indicated that Imp3, Imp9, Imp-α1, Imp-α5 and Imp-β1 do not act synergistically with Imp7. Taken together, these results suggest that Imp7 plays a dominant role in regulating the nuclear translocation of p38.

### Imp7 is necessary for p38-dependent gene expression

**Fig. 5:**
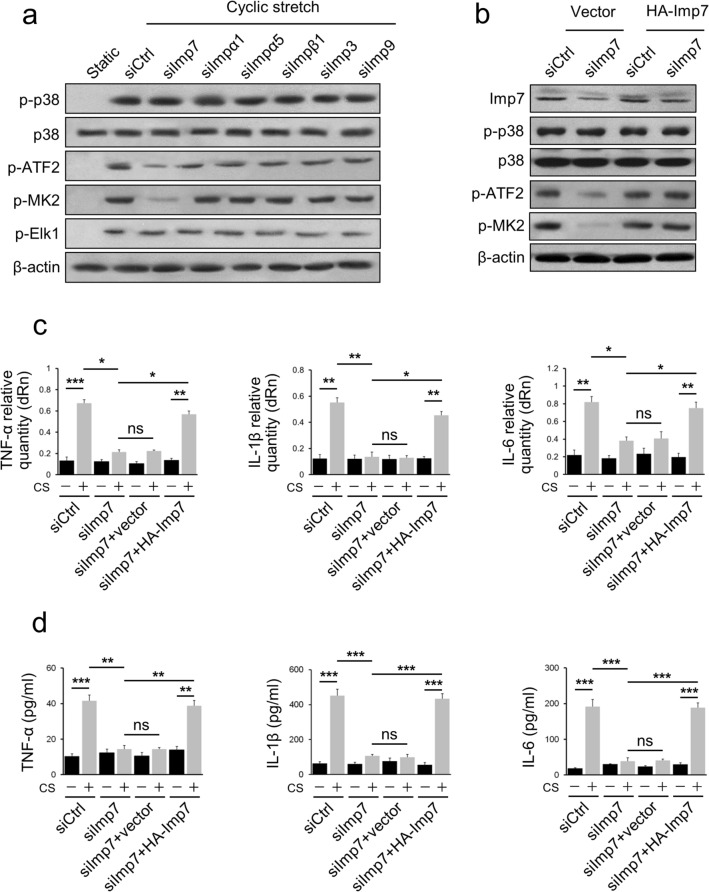
Imp7 is necessary for p38-dependent gene expression. **a** AECs were transfected with siRNA specific to Imp7 (siImp7), Imp-α1 (siImpα1), Imp-α5 (siImpα5), Imp-β1 (siImpβ1), Imp3 (siImp3), Imp9 (siImp9), or control (siCtrl), respectively, and treated with CS for 10 min, then Western blotting was performed with p-p38, p38, p-ATF2, p-MK2, p-Elk1, and β-actin antibodies. Cells that were not subjected to CS served as a negative control (static). **b** AECs were transfected with siImp7 or siCtrl, and an empty vector or HA-Imp7 were overexpressed. The cells were treated with CS for 10 min, then Western blotting was performed with Imp7, p-p38, p38, p-ATF2, p-MK2, and β-actin antibodies. **c** AECs were transfected with the siImp7 or siCtrl, and an empty vector or a HA-Imp7 were overexpressed. The cells were treated with or without CS for 10 min, and qRT-PCR was performed to check TNF-α, IL-1β, and IL-6 mRNA expression. **d** AECs were transfected with siImp7 or siCtrl, and an empty vector or HA-Imp7 were overexpressed. The cells were treated with or without CS for 10 min, and then ELISA was performed to check the levels of TNF-α, IL-1β, and IL-6 in the cell culture supernatant. Three independent experiments were analyzed. Significance: *P < 0.05, **P < 0.01, ***P < 0.001, *NS* no significance; two-tailed Student’s *t* tests

To explore the potential function of Imp7 through interaction with p38, we silenced Imp7, Imp-α1, Imp-α5, Imp-β1, Imp3, or Imp9 with specific siRNAs in AECs and observed the effect on the activation of ATF-2, MK2, and Elk1. The results showed that the knockdown of Imp7, but not other NTRs, effectively prevented CS-induced ATF-2 and MK2 but not Elk1 activation (Fig. 5a), demonstrating that the effect of Imp7 is due to its interaction with p38 rather than ERK/JNK. We further performed rescue experiments using a plasmid containing wild-type Imp7 gene (HA-Imp7) and found that HA-Imp7 transfection was sufficient to abolish the knockdown of Imp7 expression by siRNA, indicating that the siRNA is highly selective for the endogenous Imp7 gene (Fig. 5b). We also showed that exogenous Imp7 transfection successfully rescued CS-induced ATF-2 and MK2 activation when co-transfected with Imp7 siRNA, suggesting that this phenotype is directly related to p38-mediated transcriptional activation function (Fig. 5b).

The results were further validated by changes in mRNA and protein levels of proinflammatory cytokines in AECs in response to CS. As expected, CS induced increased expression of TNF-α, IL-1β, and IL-6 genes, as well as the cytokines released into the culture supernatant; however, this effect was not observed in siImp7-transfected cells (Fig. 5c, d). Notably, when Imp7 was overexpressed, the levels of both these proinflammatory genes and proteins were rescued (Fig. 5c, d).

### Administration of Imp7 siRNA nanoparticles attenuated VILI

**Fig. 6:**
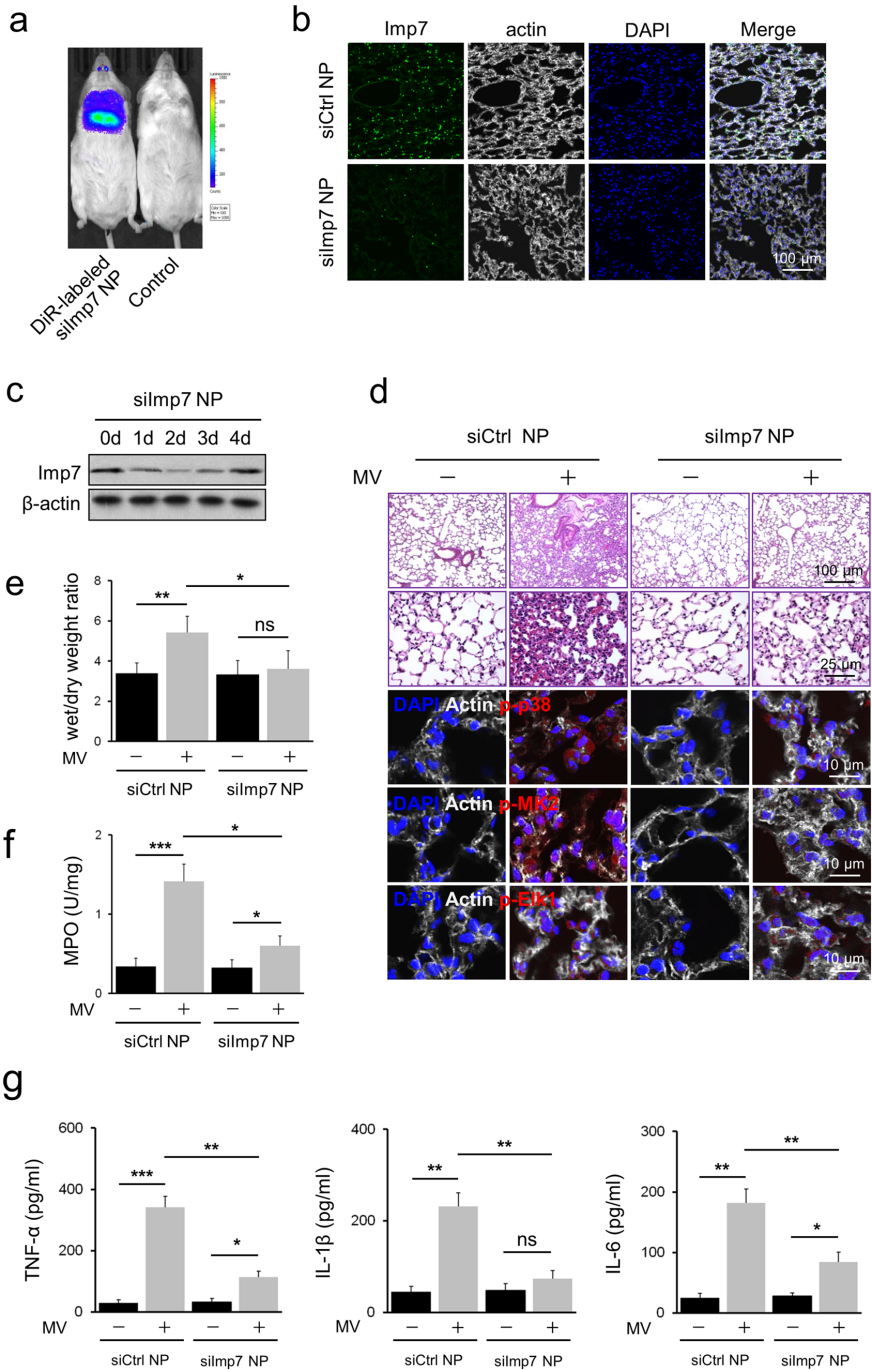
Administration of Imp7 siRNA-loaded nanoparticles attenuated VILI. **a** Representative IVIS images show the fluorescence signal was mainly concentrated in the lung in the DiR-labeled Imp7 siRNA (siImp7) nanoparticles (NP) administered group compared with the control group. **b** The mice were administered siImp7 or control siRNA (siCtrl) NP intratracheally (500 µL), and immunostaining was performed with Imp7 (green), F-actin (white) antibodies, and counterstained with DAPI to detect nuclei (blue). **c** The mice were administered siImp7 NP intratracheally (500 µL), and Western blotting was performed at the indicated time point with Imp7 and β-actin antibodies. **d**–**g** Mice were administered siImp7 or siCtrl NP in PBS (500 µL) intratracheally, and were treated with or without mechanical ventilation (MV) for 4 h with a tidal volume of 15 ml/kg and a respiratory rate of 100 breaths/min. **d** Mice lungs were sectioned and stained with H&E, and tissue damage induced by MV was assessed by light microscopy. Immunostaining was performed with p-p38 (line3, red), p-MK2 (line4, red), p-Elk1 (line5, red), F-actin (white) antibodies, and counterstained with DAPI to detect nuclei (blue). **e** The wet/dry weight ratio was calculated as the ratio of the wet weight to the dry weight. **f** MPO activity in lung tissue lysates was determined by ELISA. **g** The levels of TNF-α, IL-1β, and IL-6 in BALF were determined by ELISA. Three independent experiments were analyzed. Significance: *P < 0.05, **P < 0.01, ***P < 0.001, *NS* no significance; two-tailed Student’s *t* tests. (color figure online)

**Fig. 7:**
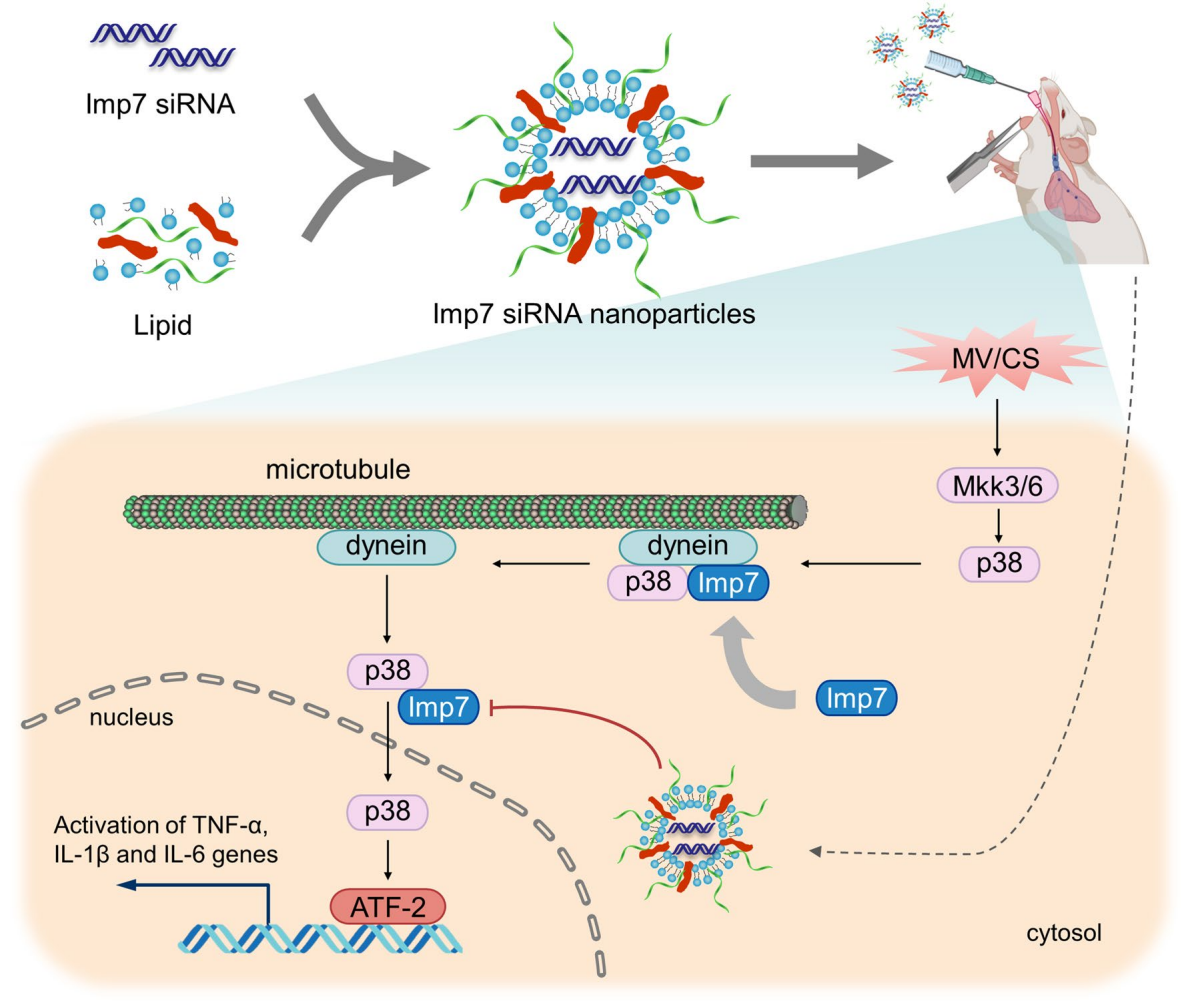
Model depicting the mechanism by which targeting the nuclear translocation of p38 by Imp7 inhibition attenuates inflammatory response in response to mechanical stretch

The above results prompted us to evaluate the therapeutic effect of Imp7 depletion in vivo. We generated lipid-based nanoparticles loaded with Imp7 siRNA (Fig. 7). For in vivo optical imaging, DiR-labeled Imp7 siRNA nanoparticles were injected intratracheally into the mice. The results showed that a considerable fluorescent signal was detected in the lungs of DiR-labeled Imp7 siRNA nanoparticles injected animals. In contrast, no signal was observed in the control group (Fig. 6a). Then, Imp7 expression was examined after delivery of the nanoparticles. Notably, a significant decrease in Imp7 expression was observed (Fig. 6b). To determine the appropriate time window to initiate MV, we assessed changes in Imp7 expression in the lung following nanoparticles administration. There was a significant decrease on day 2, and the expression gradually increased and recovered on day 4 (Fig. 6c).

Finally, mice were administered Imp7 siRNA or control nanoparticles and mechanically ventilated on day 2. The therapeutic effect of Imp7 siRNA nanoparticles was validated in VILI mice, as indicated by attenuated histological damage (Fig. 6d) and reduced lung wet/dry weight ratio (Fig. 6e). Furthermore, mice administered Imp7 siRNA nanoparticles exhibited significant reductions in MPO activity (Fig. 6f) and TNF-α, IL-1β, and IL-6 levels in BALF (Fig. 6g). We also stained p-p38, p-MK2 and p-Elk1 in the lungs. The result showed that the Imp7 siRNA nanoparticles inhibited p38 nuclear translocation and reduced the activation of MK2, further proving the specificity of Imp7 effect was due to the p38 translocation rather than ERK/JNK (Fig. 6d). Taken together, these data highlight the therapeutic potential of Imp7 siRNA-loaded nanoparticles targeting p38 to treat VILI.

## Discussion

The ability of p38 to phosphorylate substrates in the nucleus and the role of nuclear p38 in the regulation of inflammation have focused attention on the subcellular localization of the kinase. Although it is clear that p38 shuttles to the nucleus upon stimulation, the regulatory mechanisms that govern the process have yet to be fully defined. Here, we demonstrate that CS induces phosphorylation-dependent nuclear translocation of p38, which requires the involvement of microtubules and dynein. Endogenous pull-down analysis revealed Imp7 to be a potential p38-interacting protein. Silencing Imp7 inhibited CS-induced nuclear translocation of p38 as well as cytokine release, suggesting the promise of targeting Imp7 to modulate p38-related inflammatory responses. Notably, intratracheal administration of Imp7 siRNA nanoparticles significantly attenuated lung inflammation and histological damage in a VILI mouse model (Fig. 7). Together, our study reveals a potent role of Imp7 during p38 nuclear import following CS stimulation and provides insights into the therapeutic potential of preventing p38 nuclear translocation through Imp7 inhibition in the treatment of VILI.

P38 is known for its involvement in stress signaling. Abnormal activity and dysregulation of the p38 cascade are associated with many pathologies, especially inflammation. Over the past decade, numerous studies have been devoted to investigating the role of p38 in inflammation-related diseases [36]. Like all other MAPKs, activated p38 performs its function through phosphorylation of downstream proteins. One of the most important mechanisms that determines the ability of MAPKs to phosphorylate different substrates under varying conditions or cell lines is the cellular localization of the substrates [37]. Since there are more than 150 direct substrates of p38 in the nucleus, it can translocate into the nucleus to activate different nuclear targets and execute different signaling events upon different stimulations [12]. Such an effect is supported by our findings that CS activates and induces p38 nuclear import, and inhibition of the import prevents CS-induced cytokine release and alleviates VILI, confirming the importance of p38 nuclear translocation in response to mechanical stimulation. We also showed that CS-induced nuclear import of p38 requires its phosphorylation by the upstream kinase MKK3/6, but is independent of its catalytic activity. These results are consistent with previous studies applying other stimuli [17], suggesting that phosphorylation of p38 may induce conformational changes, unmasking a domain that enables it to interact with other proteins, which may mediate its shuttling into the nucleus, and this change is part of a general response to environmental stress.

The importance of nuclear p38 in regulating gene expression has prompted extensive research on the molecular mechanisms of its nuclear shuttling. Increasing evidence demonstrated that p38 is localized in the cytoplasm of resting cells due to interactions with various anchoring proteins such as phosphatases PTP-SL [38], keratins [23], and others [39]. Upon stimulation, most p38 molecules detach from their cytoplasmic anchors and rapidly translocate into the nucleus via the nuclear pore [40]. However, how activated p38 moves from its anchor point to the nuclear pore remains poorly understood. It is conceivable that the involvement of the cytoskeletal system might facilitate this process, as the association between MAPK components and the microtubule and actin filament network has long been observed [41–44]. In fact, microfilament, microtubule, and dynein have been reported to mediate multiple stimuli-induced nuclear translocation of p38 [17, 23, 45]. Here, we found that microtubules, but not microfilaments, are critical in CS-induced nuclear import of p38 and its subsequent phosphorylation of ATF-2. In the cases we tested, only treatment with the microtubule depolymerizing agent nocodazole reduced the extent of nuclear accumulation of p38. This process was also blocked after inhibiting dynein function with ciliobrevin D. Thus, CS-induced nuclear import of p38 can be enhanced by allowing the protein to utilize microtubule motor proteins to move along microtubules to the nucleus. The discrepancy between our results and those involving actin may be due to the use of different stimuli and cell types.

As mentioned above, p38 mainly distributes in the cytoplasm of resting cells and translocates into the nucleus upon activation to access its nuclear substrate. With the disappearance of the stimulation, inactivated p38 is exported to the cytoplasm to receive other stimulations [16, 21, 45]. Our study shows that CS stimulation rapidly induces nuclear translocation of p38 to mediate cytokine upregulation, highlighting the importance of p38 localization for its biological function. Like most other MAPKs, p38 does not contain canonical NLS. Furthermore, it does not appear to interact with classical importin-α/β or use passive diffusion for nuclear shuttling [12]. Therefore, NLS-containing proteins other than importin-α/β might escort p38 into the nucleus upon mechanical stretch stimulation. To explore potential partners, we performed endogenous pull-down assays. Importin-7, a member of a group of β-like importins (importin 2 to 13, also known as karyopherin βs [46, 47]), was identified as a CS-induced p38-interacting protein.

The role of Imp7 in the nuclear translocation of NLS-deficient signaling proteins seems to be essential but poorly investigated [47, 48]. Imp7 has been reported to be involved in the nuclear import of several cargoes, including ribosomal proteins, histone H1, a few kinases (SYK, ERK2, and MEK1), and the transcription factors SMAD3 and EGR1 [49–55]. Imp7 is also known to import activated MAPKs into the nucleus of Drosophila and vertebrates [39, 53, 56]. In fact, several recent studies have demonstrated the importance of Imp7 in regulating p38 shuttling. Three β-like import proteins, Imp3, 7, and 9, were reported to mediate the nuclear translocation of p38 [25]. It was further established that p38 interacted with Imp7 through its N-terminus, and a peptide designed to target this specific site prevented this interaction, thereby inhibiting its nuclear translocation upon stimulation [18]. Other studies demonstrated that upon mechanical stimulation, the Hippo pathway effector YAP ensures its nuclear translocation by monopolizing Imp7 [34]. Thus, the interaction between Imp7 and its cargoes ensures the nuclear translocation of these cargoes, which might provide a unique mechanism of nuclear import regulation in response to mechanical signals. Here we have shown yet another canonical NLS-independent mechanism for the nuclear translocation of p38 induced by mechanical stretch. In this model, p38 translocated into the nucleus along microtubules with the assistance of Imp7. Therefore, Imp7 should be involved in multiple mechanisms and interact with different cargoes to facilitate the nuclear translocation of proteins induced by various stimuli. In the present study, mechanical stretch-induced p38 nuclear import did not require Imp3 and Imp9, which have been demonstrated to mediate the nuclear translocation of p38 [25]. The discrepancy between our results and those from Zehorai et al. [25] may be owing to the varied stimuli and cell types used. We also show here that siImp7 inhibited the activation of MK2, further proving that the effects of Imp7 are due to its interaction with p38. Interestingly, while p38 is capable of activating MK2 in the nucleus, the cellular location of p38 itself has been shown to be controlled by MK2 and possibly MK5 [57]. Following phosphorylation and activation of MK2, nuclear p38 is exported to the cytoplasm in a complex with MK2 [13]. Although MK2 and MK5 have not yet been proven to contribute to mechanical stretch-induced p38 nuclear export, further research will be required to understand whether this mechanism is conserved under different conditions.

Given the central role of p38 in cellular responses to inflammatory stress, it is clear that kinase-specific inhibitors should be a beneficial therapeutic approach. In fact, over the past decade, more than twenty p38-specific inhibitors have been developed and demonstrated to be effective in preclinical studies, showing good tolerability and efficacy in several mouse models [58]. However, the effects were much less favorable in clinical trials. The relatively high toxicity and lack of durable therapeutic effects of these drugs exclude their use in any inflammation-related disease [12, 20, 59]. Therefore, we aimed to develop a new strategy targeting nuclear translocation of p38, which contains fewer side effects and toxicity than currently developed strategies [19]. Here, we found that Imp7 siRNA completely prevented CS-induced nuclear import of p38 and subsequent phosphorylation of transcription factors. It has beneficial effects as a protective anti-inflammatory agent that inhibits the production of proinflammatory cytokines. Importantly, Imp7 siRNA-loaded nanoparticles attenuated lung inflammation and tissue damage in a VILI mouse model. This activity is mainly attributed to the effect of the nanoparticles on preventing nuclear import of p38. Therefore, the animal model used here supports the use of inhibition of p38 nuclear translocation as a new drug target for VILI, and inhibitors of the kinase translocation should be further developed for clinical use.

## Conclusions

In summary, we reveal a potent role of importin-7 in p38 nuclear import in response to mechanical stretch and provide new insights into the therapeutic potential of preventing p38 nuclear translocation in the treatment of ventilator-induced lung injury.

## Data Availability

All data generated or analyzed during this study are included in this published article.
